# Glycosphingolipid expression at breast cancer stem cells after novel thieno[2,3-*b*]pyridine anticancer compound treatment

**DOI:** 10.1038/s41598-020-68516-y

**Published:** 2020-07-17

**Authors:** Sandra Marijan, Anita Markotić, Angela Mastelić, Nikolina Režić-Mužinić, Lisa Ivy Pilkington, Johannes Reynisson, Vedrana Čikeš Čulić

**Affiliations:** 1 Department of Medical Chemistry and Biochemistry, University of Split School of Medicine, 21000 Split, Croatia; 2 School of Chemical Sciences, The University of Auckland, Auckland, New Zealand; 3 School of Pharmacy and Bioengineering, Keele University, Staffordshire, UK

**Keywords:** Drug development, Molecular medicine

## Abstract

Glycosphingolipid expression differs between human breast cancer stem cells (CSC) and cancer non-stem cells (non-CSC). We performed studies of viability, type of cell death, cancer stem cell percent and glycosphingolipid expression on CSC and non-CSC after treatment of MDA-MB-231 and MDA-MB-453 triple-negative breast cancer cells with a newly developed thienopyridine anticancer compound (3-amino-*N*-(3-chloro-2-methylphenyl)-5-oxo-5,6,7,8-tetrahydrothieno[2,3-*b*]quinoline-2-carboxamide, **1**). Compound **1** was cytotoxic for both breast cancer cell lines and the majority of cells died by treatment-induced apoptosis. The percent of cancer stem cells and number of formed mammospheres was significantly lower. Glycosphingolipids IV^6^Neu5Ac-nLc_4_Cer and GalNAc-GM1b (IV^3^Neu5Ac-Gg5Cer) not reported previously, were identified in both CSCs and non-CSCs. IV^6^Neu5Ac-nLc_4_Cer had increased expression in both CSCs and non-CSCs of both cell lines after the treatment with **1**, while GM3 (II^3^Neu5Ac-LacCer) had increased expression only on both cell subpopulations in MDA-MB-231 cell line. GalNAc-GM1b, Gb_4_Cer (GalNAcβ1-3Galα1-4Galβ1-4Glcβ1-1Cer) and GM2 (II^3^Neu5Ac-GalNAcβ1-4Galβ1-4Glcβ1-1Cer) were increased only in CSCs of both cell lines while GD3 was decreased in CSC of MDA-MB-231 cell line. Due to its effect in reducing the percentage of cancer stem cells and number of mammospheres, and its influence upon several glycosphingolipid expressions, it can be concluded that compound **1** deserves attention as a potential new drug for triple-negative breast cancer therapy.

## Introduction

The thieno[2,3-*b*]pyridines were initially discovered as potential inhibitors of phospholipase C (PLC) isoforms by virtual high throughput screen (vHTS)^1^. Recently, we described glycoconjugate GM3 and CD15s expression in MDA-MB-231 triple negative breast cancer stem cell subpopulation cultured with 3-amino-5-oxo-*N*-naphthyl-5,6,7,8-tetrahydrothieno[2,3-*b*]quinoline-2-carboxamide, which was developed as a putative PLC inhibitor. A close structural analogue of 3-amino-*N*-(3-chloro-2-methylphenyl)-5-oxo-5,6,7,8-tetrahydrothieno[2,3-*b*]quinoline-2-carboxamide, or compound **1**^2^ was chosen for this study due to its enhanced potency against the MDA-MB-231 cell line and its mechanism of action has been investigated^3,4^. Due to their ability to self-renew and to regenerate the primary tumour phenotypic heterogeneity, cancer stem cells are important therapeutical targets^5^. CSCs are defined with their CD44^+^/CD24^−^ or CD133^+^ phenotype^6^. It is believed that CSCs are involved in therapy resistance in various cancers, including triple-negative breast cancers, i.e., breast cancers that do not express the genes for estrogen receptor, progesterone receptor and the human epidermal growth factor receptor-2^7^.

Glycosphingolipids (GSLs), consisting of a hydrophobic ceramide and hydrophilic carbohydrate residues, are an important component of cell plasma membranes. They regulate numerous cellular processes like adhesion, proliferation, apoptosis, recognition, modulation of signal transduction pathways and cancer metastasizing^8,9^. GSLs are classified based on their structure. Gangliosides have been characterized by the presence of a common core structure Galβ1-4Glcβ1-1Cer and/or ganglio-*N*-tetraosyl core (Galβ1-3GalNAcβ1-4Galβ1-4Glcβ1-1Cer), and one or two α2-3NeuAc linked to internal or terminal Gal, or both^10^. Due to their sialic acid content (NeuAc), gangliosides are acidic GSLs. In addition to gangliosides with ganglio-*N*-tetraosyl core, neolacto-series gangliosides were described, with a Galβ1-4GlcNAcβ1-3Galβ1-4Glcβ1-1Cer core structure. They are terminally α2-3 or α2-6-sialylated, forming IV^3^Neu5Ac-nLc_4_Cer, and IV^6^Neu5Ac-nLc_4_Cer gangliosides, respectively^11,12^. Globo-series GSLs are a major component of human erythrocytes, termed “globoside” since its representative, Gb_4_Cer was obtained as a globular precipitate^10^. Globotriaosylceramide, Gb_3_, was found to have the structure Galα1-4Galβ1-4Glcβ1-Cer, and this structure is the inner core of all globo-series GSLs^13,14^. Liang et al*.* described greatly reduced levels of Fuc(n)Lc_4_Cer and Gb_3_Cer, and much higher levels of GD2 (Galβ3GalNAcβ3Galα4Galβ4GlcβCer), GD3 (II^3^(Neu5Ac)_2_-LacCer), GM2, and GD1a in breast CSCs in comparison to cancer non-stem cells^15^. Approximately 50% of invasive ductal carcinomas overexpress GD3, 9-*O*-acetyl-GD3, and 9-*O*-acetyl-GT3^16^. GD2^+^ subpopulation shows more mesenchymal stem cell features in breast phyllodes tumors^17^. Gb5 (GalNAcβ4Galβ4GlcβCer) is a potential marker of breast CSCs^18^. During induced epithelial–mesenchymal transition, Gg4 (gangliotetraosylceramide) and its synthase B3GALT4 are significantly reduced^19^.

Considering the role of CSCs in tumor relapse and resistance, the aim of this study was to investigate the effect of newly synthesized thieno[2,3-*b*]pyridine anticancer agent **1** on CSC glycosphingolipid expression. Six gangliosides GM3, GD3, GM2, GalNacGM1b, IV^3^Neu5Ac-nLc_4_Cer, and IV^6^Neu5Ac-nLc_4_Cer and three neutral GSLs (Gg_3_Cer, Gb_4_Cer, and nLc_4_Cer) were examined. GSL expression was compared between CSCs and non-CSCs. Cell metabolism and the type of cell death after administration of derivative **1** were assessed using the MTT (3-(4,5-dimethylthiazolyl-2)-2,5-diphenyltetrazolium bromide) assay and double cell staining (Annexin-V-Fluorescein isothiocyanate (FITC) and propidium iodide (PI)), respectively. The mammosphere formation assay has been used for determination of cancer stem cell activities in breast cancer cell lines^20^. According to Croker et al.^21^, MDA-MB-231 population consist of 80% CSCs (CD44^+^CD24^−^). In addition to this canonical lethal CSC-like MDA-MB-231, non-stem breast cancer MDA-MB-453 cells^22^ were studied in their response to new inhibitor.

## Methods

### Chemistry and cell line

**Figure 1:**
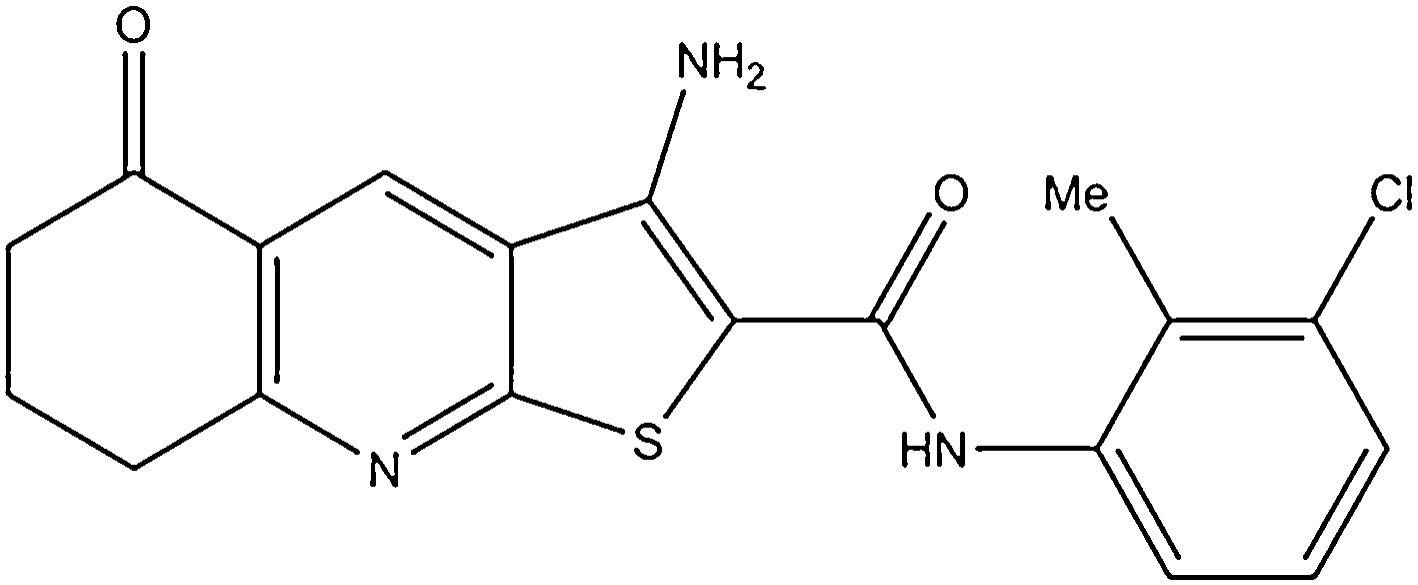
The structure of the newly synthesized anticancer agent (compound **1**). *Note* Compound 1, 3-amino-5-oxo-*N*-naphthyl-5,6,7,8-tetrahydrothieno[2,3-*b*] quinoline-2-carboxamide.

3-Amino-*N*-(3-chloro-2-methylphenyl)-5-oxo-5,6,7,8-tetrahydrothieno[2,3-b]quinoline-2-carboxamide (compound **1**) (Fig. 1) was dissolved in dimethyl sulfoxide (DMSO). Cancer cell lines MDA-MB-231 and MDA-MB-453 were grown in a humidified incubator at 37 °C and 5% CO_2_ in Dulbecco’s Modified Eagle Medium (DMEM, Sigma-Aldrich, Steinheim, Germany) containing 10% fetal bovine serum and 1% antibiotics.

### Cytotoxic activity assay

Cell metabolism was measured with MTT to estimate cell viability^23^. Equal numbers of cells were plated in five replicates and allowed to attach overnight. Cells were then treated with complete media or individual solutions of **1** at 50 nM, 0.25, 0.5, 1 and 5 µM in complete media, in five repetitions, for 4, 24, 48 and 72 h. Following treatment, cells were incubated with 0.5 mg/ml MTT in media for 1 h and then the media was removed and DMSO was added. Absorbance was measured at 570 nm^24^.

### Flow cytometric analyses

Equal numbers of cells were seeded in 6-well plates and treated with 2 µM **1** and then analysed for apoptosis. After treatment with **1**, the cells were trypsinized, washed with phosphate buffered saline (PBS) and resuspended in 100 µl of the binding buffer containing 5 µl Annexin-V-FITC and/or 5 µl of PI (Annexin-V-FITC Apoptosis Detection Kit I, BD Biosciences). The cells were incubated for 15 min at room temperature in the dark and thereafter analysed by flow cytometry (BD Accuri C6, BD Biosciences). The percentages of apoptotic cells (Annexin-V positive cells) were analysed using the FlowLogic Software (Inivai) and presented as mean ± standard deviation (SD).

MDA-MB-231 cells treated with **1** for 48 h, as well as the controls, were stained with anti-CD44-FITC (BD Biosciences), anti-CD24-phycoerythrin (PE, eBioscience, Inc. San Diego, CA, USA) and anti-GSL antibodies. The primary antibodies against GM3 (mouse IgM) and GD3 (mouse IgG3) were from Cosmo Bio Co. (Tokyo, Japan) and produced by laboratory of Dr. J. Müthing, respectively^25^. All other anti-GSL antibodies (against Gb_4_Cer, nLc_4_Cer, IV^3^Neu5Ac-nLc_4_Cer, IV^6^Neu5Ac-nLc_4_Cer, GM2, Gg_3_Cer (gangliotriaosylceramide, GalNAcβ1-4Galβ1-4Glcβ1-1Cer) and GalNAc-GM1b) were chicken polyclonal antibodies being produced and characterized by the laboratory of Dr. J. Müthing^26^. Binding of primary anti-GSL antibodies was detected with secondary antibodies conjugated with eFluor 660 fluorochrome (Abcam).

In addition to three antibodies used for MDA-MB-231 cells, MDA-MB-453 cells were stained with anti-CD133-PE/Cy7 (BioLegends, San Diego, USA).

Data acquisition of triple and fourfold stained samples was performed on a BD Accuri 6 cytometer and analysed using the FlowLogic Software. CD44^+^ cells and CD133^+^ were gated and CSC were determined. Glycosphingolipids Gb_4_Cer, nLc_4_Cer, IV^3^Neu5Ac-nLc_4_Cer, IV^6^Neu5Ac-nLc_4_Cer, GM3, GD3, GM2, Gg_3_Cer and GalNAc-GM1b were determined on CSCs (CD44^+^CD24^−^) and non-CSCs (CD44^−^/CD24^+^, CD44^+^CD24^+^ and CD44^−^/CD24^−^) in the MDA-MB-231 and CSCs (CD133^+^) and non-CSCs (CD133^−^) in the MDA-MB-453 cell line.

### Mammosphere forming assay

Cells derived from MDA-MB-231 and MDA-MB-453 cell lines were plated in 6-well low attachment suspension culture plates (Corning® Costar® Ultra-Low Attachment Multiple Well Plate, Thermo Fisher Scientific, Waltham, MA, USA) at a density of 3.5 × 10^4^ viable cells/well. Cells were grown in 2 ml MammoCult Medium Human Kit, supplemented with Proliferation Supplement 0.1 mg/ml, Heparin Solution 4 µg/ml, Hydrocortisone Stock Solution 0.48 µg/ml (all StemCell Technologies, Vancouver, Canada) and antibiotics (1% penicillin/streptomycin, Sigma-Aldrich, Steinheim, Germany). After 7 days of incubation, mammospheres larger than 50 μm were counted with an Motic AE31E Inverted Microscope (Thermo Fisher Scientific, Waltham, MA, USA) and pictured with Industrial Digital Camera (Lacerta GmbH, Austria).

### Statistical analysis

For statistical analyses t-test with unequal variances, one-way ANOVA followed by post-hoc Tukey test or Kruskal–Wallis followed by Dunn’s post-hoc test was performed using statistical software GraphPad Prism 7.0 (San Diego, CA, USA) with the significance set at *P* < 0.05.

### Ethics approval and consent to participate

Not applicable.

### Consent for publication

Not applicable.

## Results

### Compound 1—cytotoxicity

**Figure 2:**
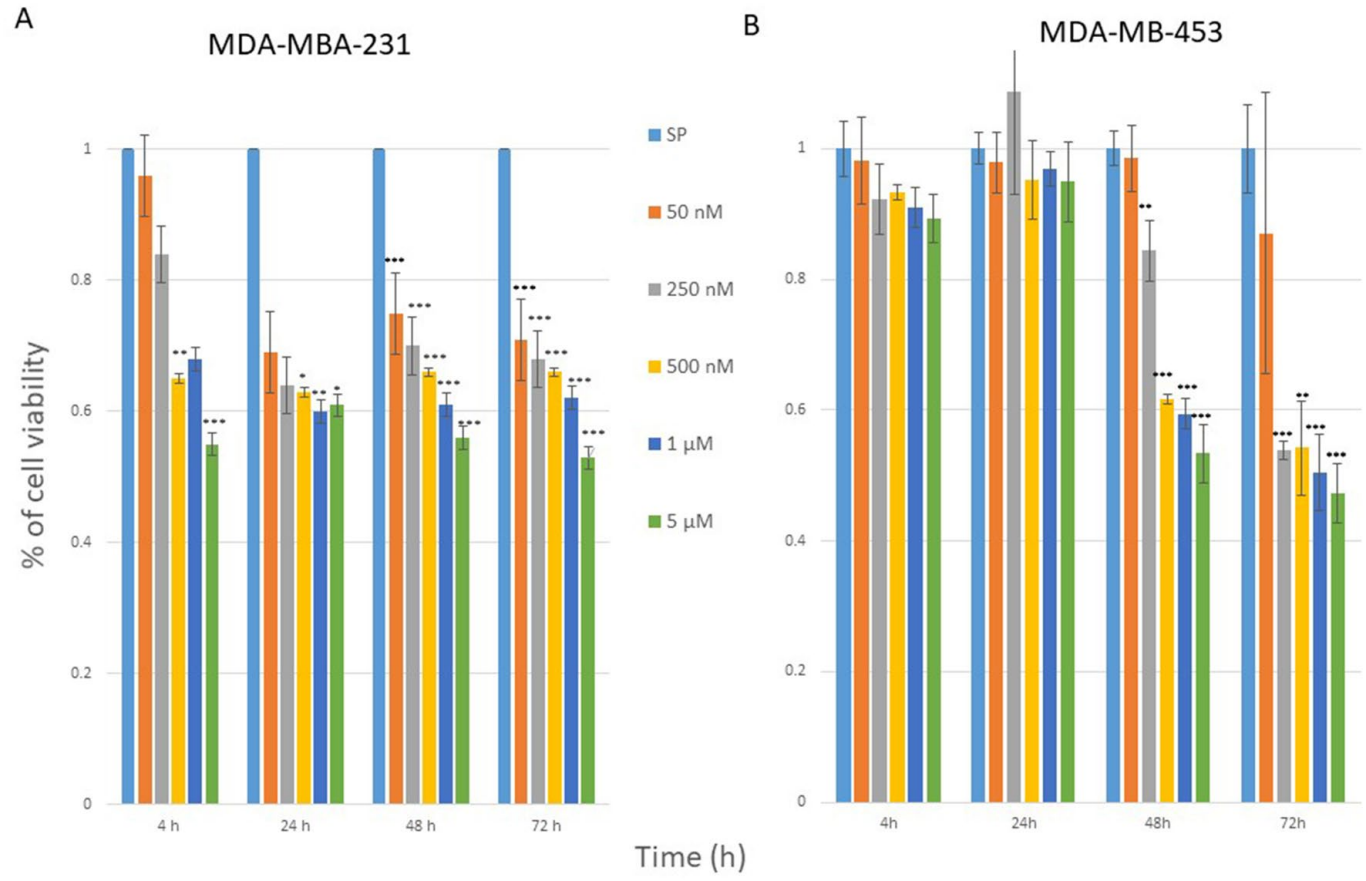
Cell viability after compound **1** treatment. *Notes* Cells were treated with a different concentration of compound **1** for 4, 24, 48 and 72 h in the MDA-MB-231 (**A**) and in the MDA-MB-453 cell line (**B**) and cell metabolism evaluated by the 3-(4,5-dimethylthiazolyl-2)-2,5-diphenyltetrazolium bromide (MTT) assay. Data are expressed as a mean from experiment performed in triplicate ± SD. Columns, mean of viable cells; bars, SD (standard deviation); *P < 0.05; **P < 0.01; ***P < 0.001. *SD* standard deviation.

Cell viabilities, after 4, 24, 48 and 72 h treatment with **1**, detected with the MTT assay are shown in Fig. 2. In the MDA-MB-231 cell line Compound **1** was shown to be cytotoxic in 0.5 µM concentration after only 4 h of treatment and ten times lower concentration (50 nM) resulted in cytotoxicity after 48 h. But maximal cytotoxicity was only achieved for 47% of the cells, 72 h after treatment with 5 µM of **1** (Fig. 2A). In the MDA-MB-453 cell line, concentration of 250 nM of **1** showed cytotoxicity after 48 h and the maximum of cytotoxic effect was after 72 h after treatment with 5 µM of **1** (Fig. 2B).

### Compound 1—mechanism of cell death

**Figure 3:**
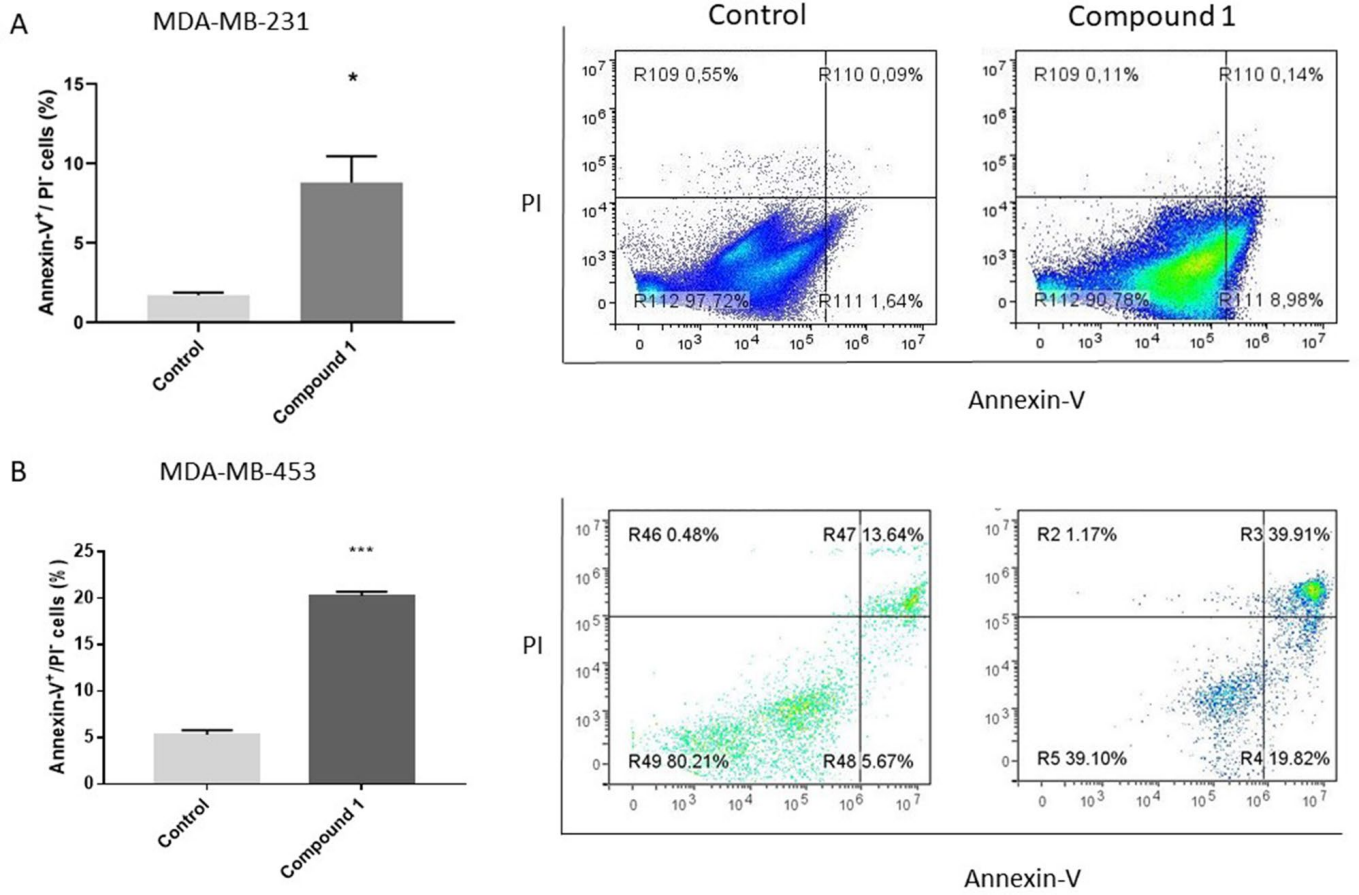
Apoptosis after compound **1** treatment. *Notes* Percentage and dot plots of apoptotic cells without and with **1** treatment for 48 h in the MDA-MB-231 (**A**) and in the MDA-MB-453 cell line (**B**). Data represent are expressed as a mean from experiment performed in triplicate ± SD. Columns, mean of cells; bars, SD; *P < 0.05; ***P < 0.001. *SD* standard deviation.

To determine whether the MTT findings are due to cell death or cell cycle arrest, we subsequently determined the type of cell death induced by 48 h treatment with 2 µM of compound **1**. The majority of cells died by treatment-induced apoptosis in both cell line as shown in Fig. 3. Compound **1** treated cells showed significant increase in early apoptosis (Annexin-V^+^PI^−^ subpopulation) in MDA-MB-231 and in MDA-MB-453 cells compared with non-treated cells, as shown in Fig. 3A, B.

### Mammosphere formation

**Figure 4:**
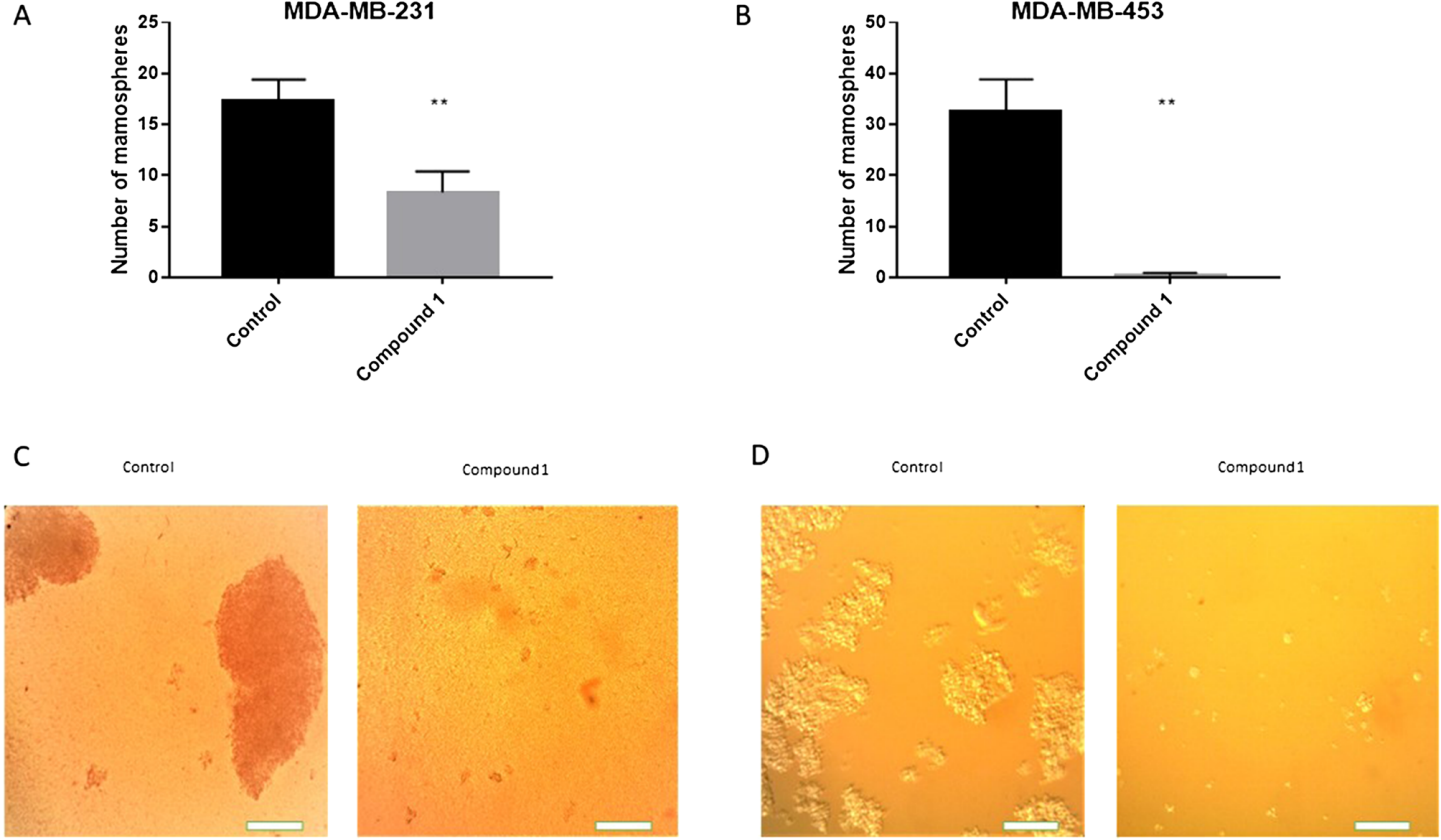
Mammosphere formation after compound **1** treatment. *Notes* Number of mammospheres without and with compound **1** treatment for 7 days in the MDA-MB-231 (**A**) and in the MDA-MB-453 cell line (**B**) and photos with × 100 magnification (scale bar, 200 μm) in the MDA-MB-231 (**C**) and in the MDA-MB-453 cell line (**D**). Mammospheres with a size over 50 μm were evaluated. Data represent are expressed as a mean from experiment performed in triplicate ± SD. Columns, mean of cells; bars, SD; **P < 0.01. *SD* standard deviation.

To determine whether the MDA-MB-231 and MDA-MB-453 cancer stem cells are sensitive to compound **1**, the number of mammospheres was counted. After treatment with compound **1** the number of mammospheres was significantly decreased in both MDA-MB-231 (Fig. 4A) and MDA-MB-453 (Fig. 4B) cell line for 52% and 99%, respectively.

### Cancer stem cells

**Figure 5:**
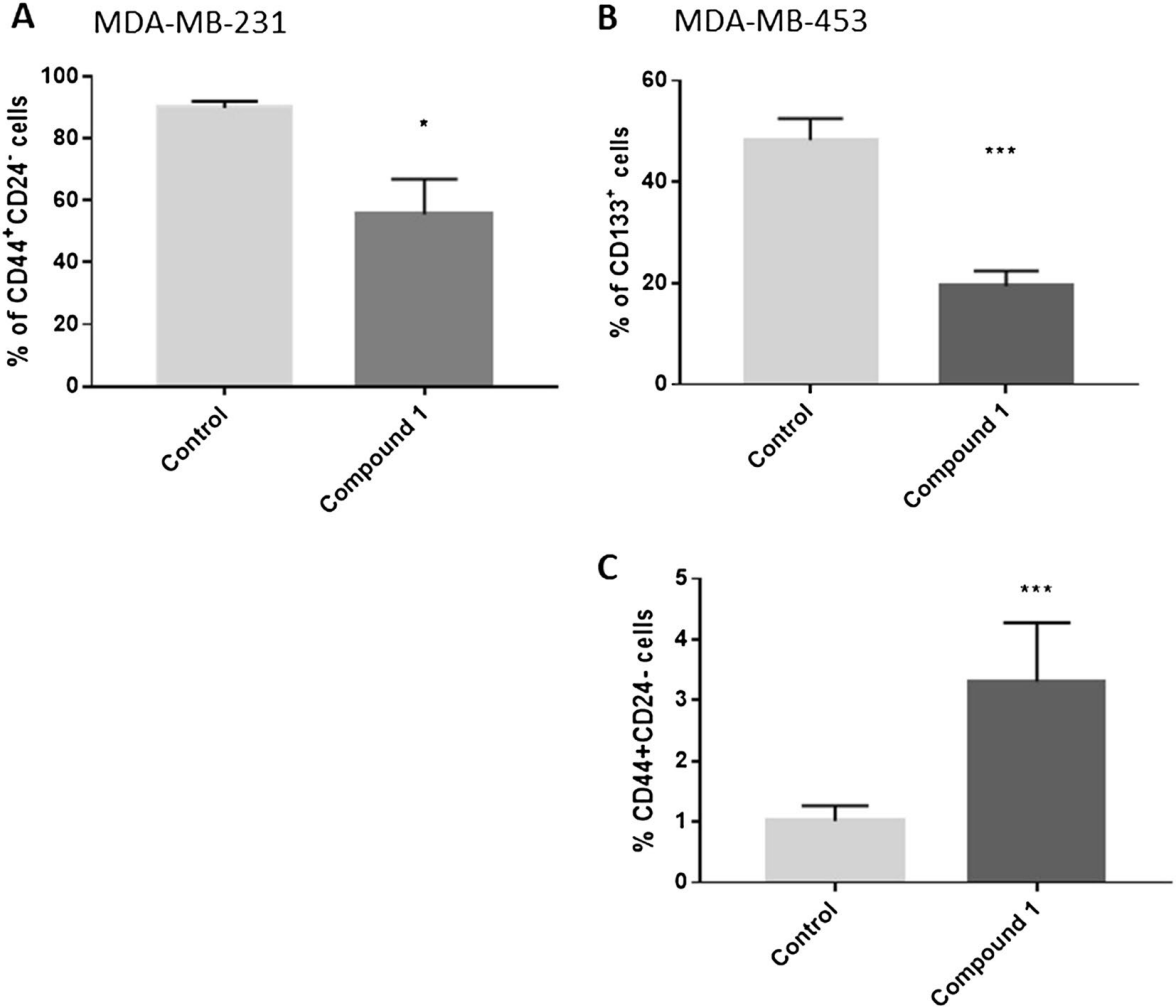
CSCs after compound **1** treatment. *Notes* Percentage of CD44^+^CD24^−^ CSCs after treatment with compound **1** for 48 h in MDA-MB 231 (**A**) and in the MDA-MB-453 cell line (**B**) and CD133^+^ CSCs in the MDA-MB-453 cell line (**C**). Data represent are expressed as a mean from experiment performed in triplicate ± SD. Columns, mean of cells; bars, SD; *P < 0.05, ***P < 0.001. *CSCs* cancer stem cells, *SD* standard deviation.

In breast cancer cell lines, such as MDA-MB-231, a subset of markers, including CD44^+^/CD24^−^ has been shown to enrich CSC^27^. Treatment with **1** resulted in a statistically significant decrease of the CD44^+^/CD24^−^ subpopulation from 89.9% (untreated control) to 55.5% (Fig. 5A)**.** In the MDA-MB-453 breast cancer cell line, expression of CD44 is very low and CD44^+^/CD24^−^ subpopulation is not considered CSC subpopulation^6^, and this subpopulation significantly increases after treatment with **1** (Fig. 5C)**.** Much more reliable marker of CSCs in the MDA-MB-453 cell line is CD133. After treatment with **1**, a significant decrease of CD133^+^ subpopulation from 48.3% in untreated control to 19.4% was obtained (Fig. 5B)**.**

### Expression of terminally sialylated gangliosides at CSCs and non-CSCs

Glycosphingolipid expression was then studied in CSC (defined as CD44^+^/CD24^−^ subpopulation in MDA-MB-231 cell line, and CD133^+^ in MDA-MB-453 cell line), with the aim of checking whether the cytotoxic effects of **1** are mediated via different GSL membrane content. In addition, GSL expression was determined in non-CSCs, being detected as three subpopulations CD44^+^/CD24^+^, CD44^−^/CD24^−^ and CD44^−^/CD24^+^ in MDA-MB-231 cell line and CD133^−^ in MDA-MB-453 cell line. Expression of each GSL per one cell is represented with geometric mean fluorescence intensity (GMI). The portion of the cells that are GSL positive is an interesting parameter, however of less impact in comparison to GMI. The terminal sugar residue of gangliosides GM3, GD3, IV^3^Neu5Ac-nLc_4_Cer and IV^6^Neu5Ac-nLc_4_Cer is sialic or *N*-acetyl-neuraminic and the last step of ganglioside GM2 and GalNAc-GM1b synthesis is transfer of GalNAc residue. Therefore, GM2 and GalNAc-GM1b expression was analysed (see next section) together with neutral GSLs Gg_3_Cer and Gb_4_Cer (globotetraosylceramide, GalNAcβ1-3Galα1-4Galβ1-4Glcβ1-1Cer), that also contain terminal GalNAc residue.

**Figure 6:**
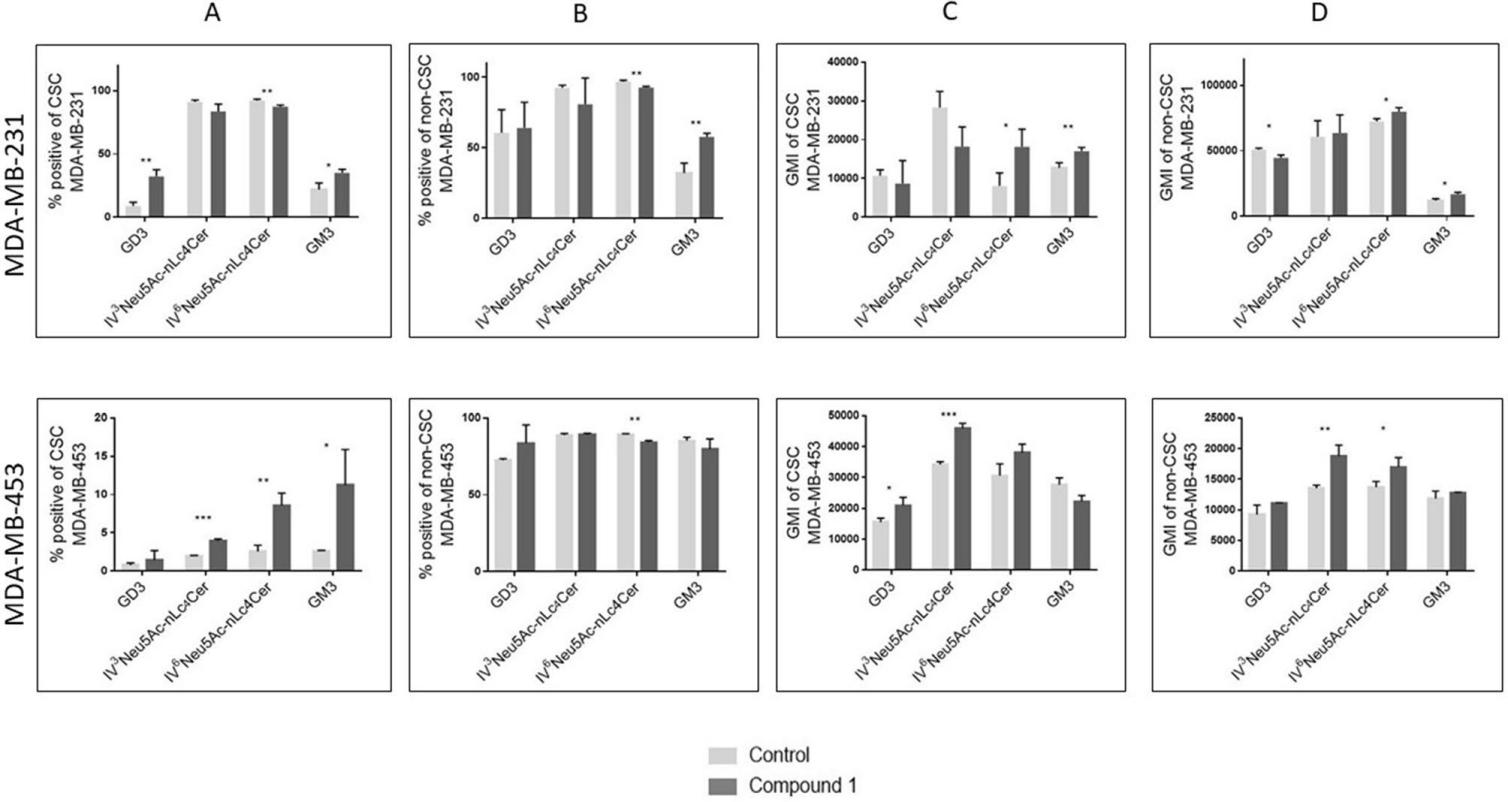
Percentage and geometric mean fluorescence intensity of terminally sialylated ganglioside positive cell subpopulations. *Notes* Percentage of CSCs in the MDA-MB-231 and in the MDA-MB-453 (**A**) and non-CSCs in MDA-MB 231 and in the MDA-MB-453 cell lines (**B**). Geometric mean fluorescence intensity of CSCs in MDA-MB 231 and in the MDA-MB-453 (**C**) and non-CSCs in the MDA-MB-231 and in MDA-MB-453 cell lines after treatment with compound 1 in duration of 48 h. Data are expressed as a mean from experiment performed in triplicate ± SD. Columns, mean of viable cells; bars, SD; *P < 0.05; **P < 0.01, ***P < 0.001. CSC MDA-MB-231, CD44^+^CD24^−^ cells of the MDA-MB-231 cell line; CSC MDA-MB-453, CD133^+^ cells of the MDA-MB-453 cell line; non-CSC MDA-MB-231, CD44^+^/CD24^+^, CD44^−^/CD24^−^ and CD44^−^/CD24^+^ cells of the MDA-MB-231 cell line; non-CSC MDA-MB-453, CD133^−^ cells of the MDA-MB-453 cell line; Neu5Ac, *N*-acetylneuraminic acid. The designation of the gangliosides follows the IUPAC-IUB recommendations^51^ and the nomenclature of Svennerholm^52^. IV^3^Neu5Ac-nLc_4_Cer; IV^6^Neu5Ac-nLc_4_Cer; GlcNAcβ1-3Galβ1-4Glcβ1-1Cer; GM3, II^3^Neu5Ac-LacCer; GD3, II^3^(Neu5Ac)_2_-LacCer; GMI, geometric mean fluorescence intensity; SD, standard deviation.

In MDA-MB-231 cell line, the percentage of GM3 positive cells was increased, whilst IV^6^Neu5Ac-nLc_4_Cer positive cells decreased within both cell subpopulations, CSC and non-CSC (Fig. 6A, B, upper row). Only the CSC population showed an increased percentage of GD3 positive cells after treatment with compound **1** (Fig. 6A, upper row). Expression of IV^3^Neu5Ac-nLc_4_Cer was not affected by treatment with compound **1**. Compound **1** increases expression of IV^6^Neu5Ac-nLc_4_Cer in both CSC and non-CSC (Fig. 6C, upper row), while non-CSC GD3 was decreased (Fig. 6D, upper row).

In MDA-MB-453 cell line, the percentages of GM3, IV^3^Neu5Ac-nLc_4_Cer and IV^6^Neu5Ac-nLc_4_Cer positive cells were significantly increased only in CSC subpopulation, whilst IV^6^Neu5Ac-nLc_4_Cer positive cells decreased within non-CSC (Fig. 6A, B, lower row). Percentage of GD3 positive cells was not affected by treatment with compound **1** (Fig. 6A, B, lower row). Compound **1** increases expression of IV^3^Neu5Ac-nLc_4_Cer in both CSC and non-CSC cells, while expression of GD3 was increased only in CSC subpopulation, and expression of IV^6^Neu5Ac-nLc_4_Cer in non-CSC cells (Fig. 6C, D, lower row).

### Expression of gangliosides and neutral GSLs with terminal GalNAc residue at CSCs and non-CSCs

**Figure 7:**
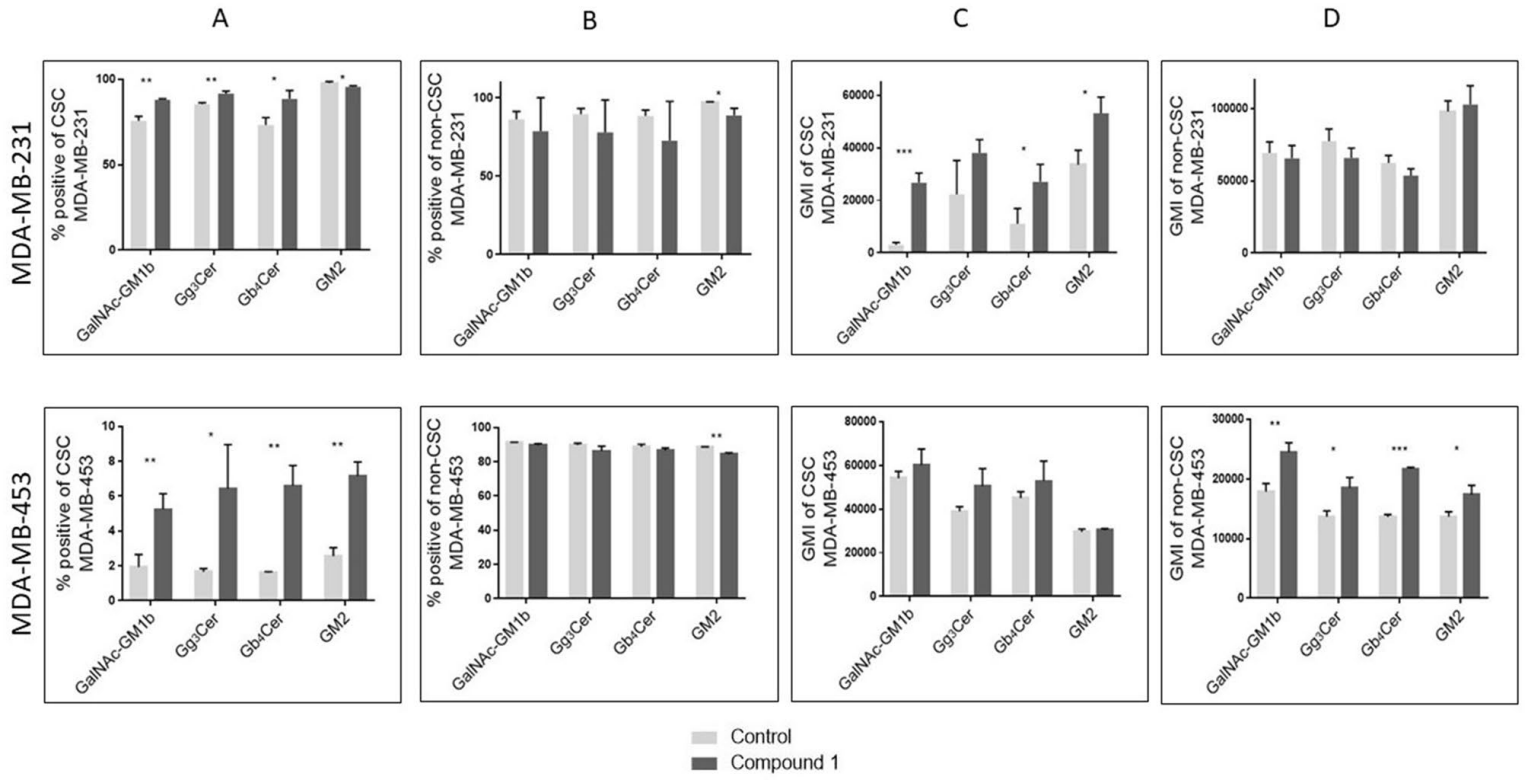
Percentage and geometric mean fluorescence intensity of cell subpopulations positive for ganglioside and neutral GSLs with terminal GalNAc residue. *Notes* Percentage of CSCs in the MDA-MB-231 and in the MDA-MB-453 (**A**) and non-CSCs in MDA-MB 231 and in the MDA-MB-453 cell lines (**B**). Geometric mean fluorescence intensity of CSCs in MDA-MB 231 and in the MDA-MB-453 (**C**) and non-CSCs in the MDA-MB-231 and in MDA-MB-453 cell lines after treatment with compound 1 in duration of 48 h. Data are expressed as a mean from experiment performed in triplicate ± SD. Columns, mean of viable cells; bars, SD; *P < 0.05; **P < 0.01, ***P < 0.001. CSC MDA-MB-231, CD44^+^CD24^−^ cells of the MDA-MB-231 cell line; CSC MDA-MB-453, CD133^+^ cells of the MDA-MB-453 cell line; non-CSC MDA-MB-231, CD44^+^/CD24^+^, CD44^−^/CD24^−^ and CD44^−^/CD24^+^ cells of the MDA-MB-231 cell line; non-CSC MDA-MB-453, CD133^−^ cells of the MDA-MB-453 cell line; globotetraosylceramide or Gb_4_Cer, GalNAcβ1-3Galα1-4Galβ1-4Glcβ1-1Cer; gangliotriaosylceramide or Gg_3_Cer, GalNAcβ1-4Galβ1-4Glcβ1-1Cer; GM2, II^3^Neu5AcGg_3_Cer; GalNAc-GM1b, IV^3^Neu5Ac-Gg_5_Cer; GM1b, IV^3^Neu5Ac-Gg_4_Cer; gangliotetraosylceramide or Gg_4_Cer, Galβ1-3GalNAcβ1-4Galβ1-4Glcβ1-1Cer; GMI, geometric mean fluorescence intensity; SD, standard deviation.

The percentage of GM2 positive cells was decreased within both cell subpopulations, CSC and non-CSC in MDA-MB-231 cell line (Fig. 7A, B, upper row), whilst only CSC populations showed an increased percentage of GalNAc-GM1b, Gg_3_Cer, and Gb_4_Cer positive cells after treatment with compound **1** (Fig. 7A, upper row). Compound **1** significantly increases the expression of GalNAc-GM1b, Gb_4_Cer and GM2 (Fig. 7C, upper row) in CSC and not significantly the expression of GM2 in non-CSC of MDA-MB-231 cell line (Fig. 7D, upper row).

In MDA-MB-453 cell line, percentages of GM2, GalNAc-GM1b, Gg_3_Cer, and Gb_4_Cer positive cells were significantly increased only in CSC subpopulation, whilst percentage of GM2 was slightly decreased in non-CSC subpopulation (Fig. 7A, B, lower row). Compound **1** significantly increases the expression of GM2, GalNAc-GM1b, Gg_3_Cer, and Gb_4_Cer in CSC subpopulation (Fig. 7D, lower row), while expression of these GSLs was not affected by treatment with compound **1** in CSC of MDA-MB-453 cell line (Fig. 7C, lower row).

### Expression of neutral GSL with terminal Gal residue at CSCs and non-CSCs

**Figure 8:**
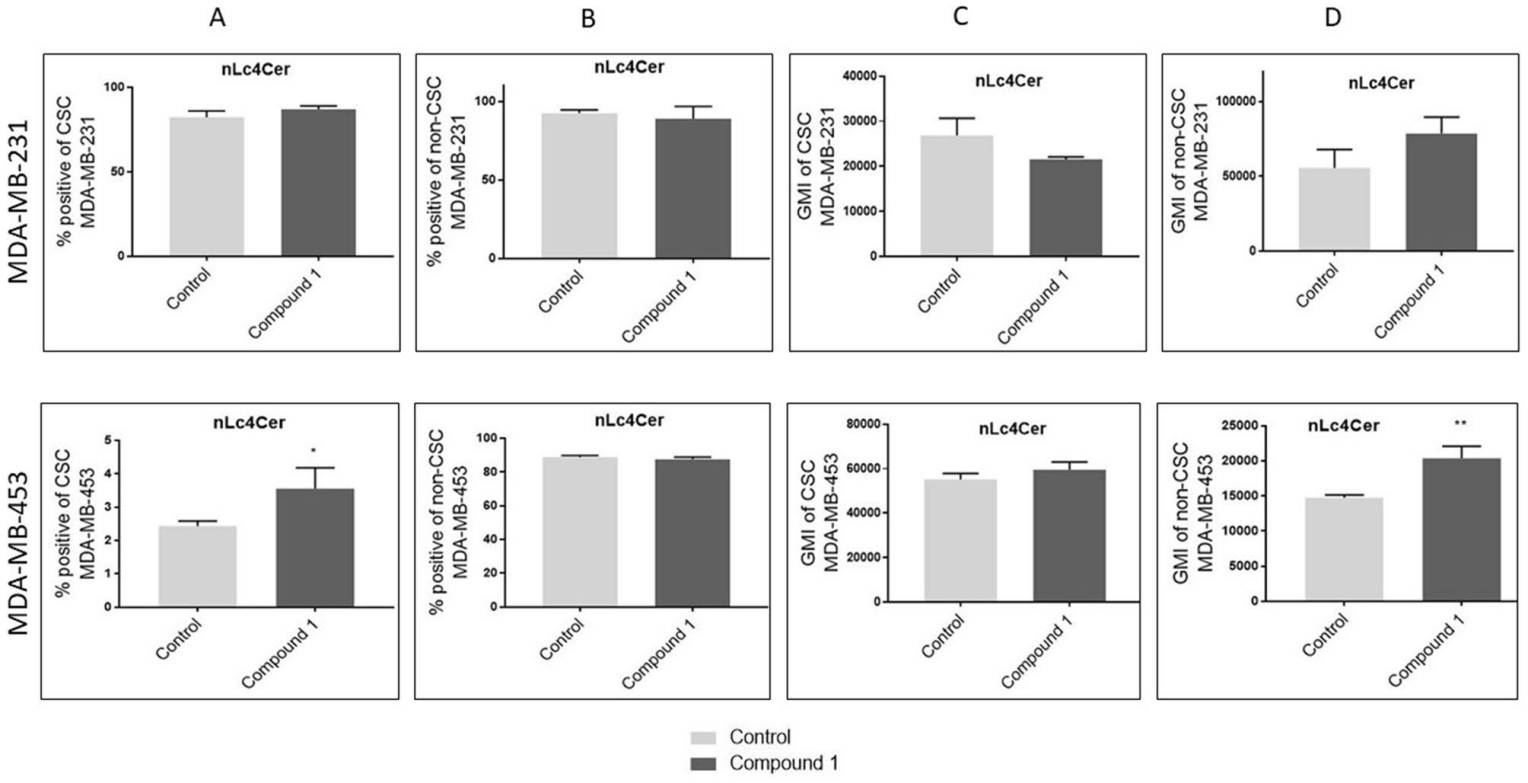
Percentage and geometric mean fluorescence intensity of cell subpopulations positive for neutral GSL with terminal Gal residue. *Notes* Percentage of CSCs in the MDA-MB-231 and in the MDA-MB-453 (**A**) and non-CSCs in MDA-MB 231 and in the MDA-MB-453 cell lines (**B**). Geometric mean fluorescence intensity of CSCs in MDA-MB 231 and in the MDA-MB-453 (**C**) and non-CSCs in the MDA-MB-231 and in MDA-MB-453 cell lines after treatment with compound 1 in duration of 48 h. Data are expressed as a mean from experiment performed in triplicate ± SD. Columns, mean of viable cells; bars, SD; *P < 0.05; **P < 0.01. CSC MDA-MB-231, CD44^+^CD24^−^ cells of the MDA-MB-231 cell line; CSC MDA-MB-453, CD133^+^ cells of the MDA-MB-453 cell line; non-CSC MDA-MB-231, CD44^+^/CD24^+^, CD44^−^/CD24^−^ and CD44^−^/CD24^+^ cells of the MDA-MB-231 cell line; non-CSC MDA-MB-453, CD133^−^ cells of the MDA-MB-453 cell line; neolactotetraosylceramide or nLc_4_Cer. GMI, geometric mean fluorescence intensity; SD, standard deviation.

There was no difference in the percentage of nLc_4_Cer positive cells in both CSC and non-CSC after treatment of MDA-MB-231 cells with compound **1** (Fig. 8A, B, upper row). Also, the expression of nLc_4_Cer was not affected by compound **1**, in both cell subpopulations of this cell line (Fig. 8C, D, upper row).

The percentage of nLc_4_Cer positive cells was significantly increased in CSC subpopulation in MDA-MB-453 cell line treated with compound **1** (Fig. 8A, lower row), and not affected in non-CSC^−^ subpopulation (Fig. 8B, lower row). The expression of nLc_4_Cer was increased in non-CSC subpopulation treated with compound **1** (Fig. 8D, lower row), while expression of nLc_4_Cer was not affected by treatment with compound **1** in CSC of MDA-MB-453 cell line (Fig. 8C, lower row).

## Discussion

We found that newly developed anticancer compound, 3-amino-*N*-(3-chloro-2-methylphenyl)-5-oxo-5,6,7,8-tetrahydrothieno[2,3-b]quinoline-2-carboxamide (compound **1**; Fig. 1) was cytotoxic for both breast MDA-MB-231 and MDA-MB-453 cancer cells. In comparison to related 3-amino-5-oxo-*N*-naphthyl-5,6,7,8-tetrahydrotieno[2,3-*b*]quinoline-2-carboxamide, reported earlier^2^, compound **1** was more cytotoxic. Halving of MDA-MB-231 cell viability was achieved with fivefold lower concentration after 4 h of treatment (5 µM compared to 25 µM). Both compounds are *not* lipophilic promiscuous inhibitors but target a specific receptor^28^. The molecular weight for compound 1 (385.867 g/mol) is in the so called ‘sweet spot’ for drug development^29^.

Determination of the type of cell death showed that **1**-induced cell death of breast cancer cells occurred mainly by apoptosis and the percentage of CSC subpopulation was significantly lower after treatment with **1**. For the first time, we report IV^6^Neu5Ac-nLc_4_Cer and GalNAc-GM1b GSL expression, in both breast CSCs and non-CSCs. After treatment with compound **1**, a significant increase in IV^6^Neu5Ac-nLc_4_Cer expression in both cell subpopulations of both MDA-MB-231 and MDA-MB-453 cell lines was observed and increase of GM3 only on both cell subpopulations in MDA-MB-231 cell line. Increase of GalNAc-GM1b, Gb_4_Cer and GM2 was only observed in CSCs of both cell lines, whilst non-CSCs of MDA-MB-231 cell line expressed lower GD3 after compound **1** treatment. Expression of GD3 on non-CSCs of MDA-MB-453 cell line was not affected by compound **1** treatment.

**Figure 9:**
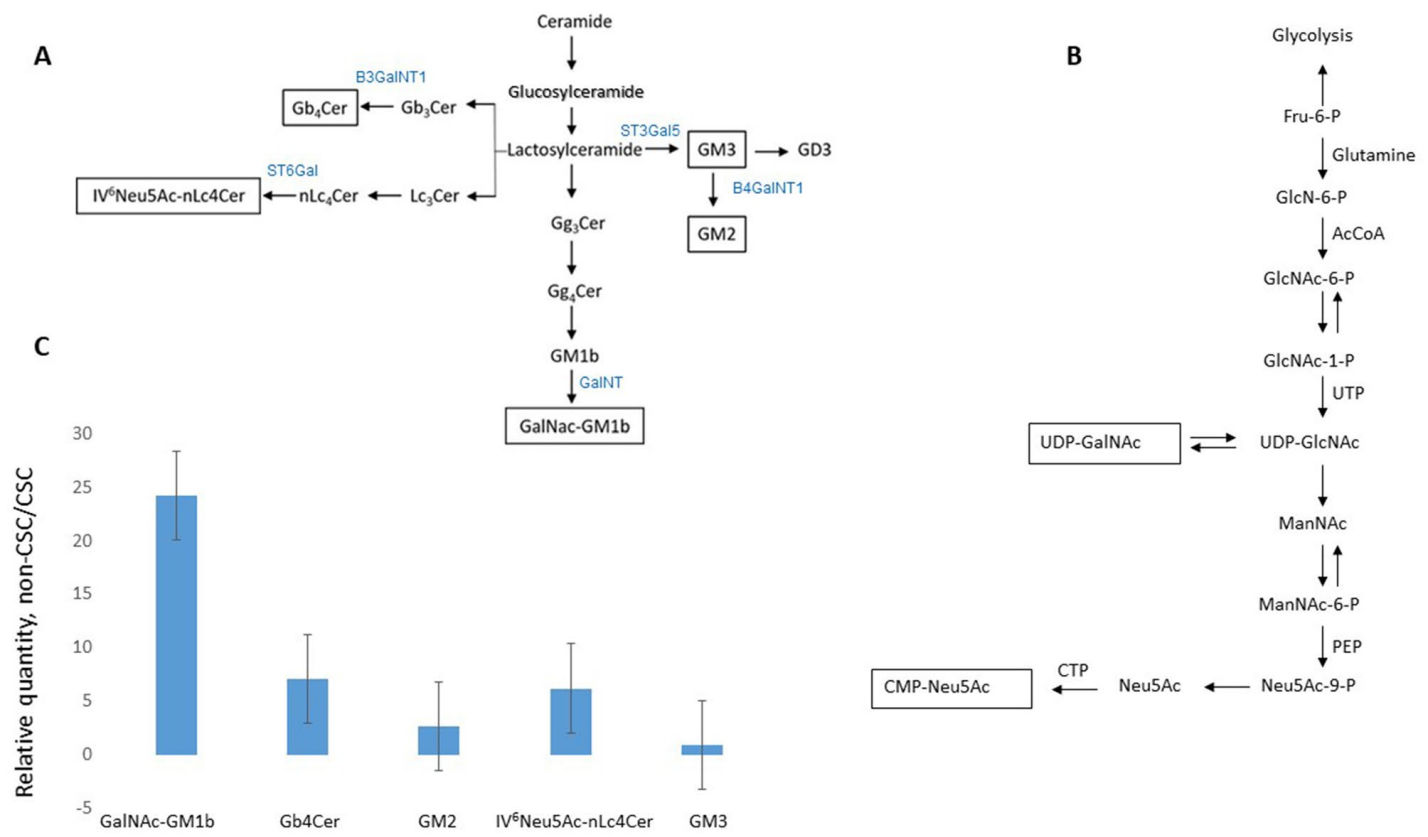
Scheme of GSL and activated sugar residue synthesis and fold changes of GSL expression in the MDA-MB-231 cell line. *Notes* Synthesis of GSLs (**A**) and of sugar residues (**B**) needed for the last step (framed at **B**) of GSL synthesis that were elevated (framed at **A**) in treated CSCs. Fold changes of GSL expression between non-treated non-CSCs and CSCs (**C**). GalNAc-GM1b, IV^3^Neu5Ac-Gg5Cer; GalNT, transferase of GalNac to GM1b; globotetraosylceramide or Gb_4_Cer, GalNAcβ1-3Galα1-4Galβ1-4Glcβ1-1Cer; B3GalNT1, transferase of GalNAc to Gb_3_Cer; GM2, II^3^Neu5Ac-GalNAcβ1-4Galβ1-4Glcβ1-1Cer; B4GalNT, transferase of GalNAc to GM3; IV^6^Neu5Ac-nLc_4_Cer; ST6Gal, sialyl transferase to nLc_4_Cer; GM3, II^3^Neu5Ac-LacCer; ST3Gal5, sialyl transferase to LacCer; Fru-6-P, fructose-6-phosphate; GlcNAc-6-P, *N*-acetylglucosamine-6-phosphate; AcCoA, Acetyl-CoA; GlcNAc-1-P, *N*-acetylglucosamine-1-phosphate; UTP, uridine-triphosphate; UDP-GlcNAc, uridine diphosphate *N*-acetylglucosamine; UDP-GalNAc, uridine 5*′*-diphospho-*N*-acetylgalactosamine; ManNAc, *N*-acetylmannosamine; ManNAc-6-P, *N*-acetylmannosamine-6-phosphate; PEP, phosphoenolpyruvate carboxylase; Neu5Ac-9-P, *N*-acetylneuraminic acid-9-phosphate; Neu5Ac, *N*-acetylneuraminic acid; CTP, cytidine triphosphate; CMP-Neu5Ac, Cytidine-5′-monophospho-*N*-acetylneuraminic acid.

Glycosphingolipids that were increased in both CSCs and non-CSCs after compound **1** treatment of MDA-MB-231 cells are acidic GSLs: gangliosides GM3 and IV^6^Neu5Ac-nLc_4_Cer. Sialic or *N*-acetyl-neuraminic acid (Neu5Ac) is added in the last step of their synthesis (Fig. 9A). The last step of GalNAc-GM1b, Gb_4_Cer and GM2 synthesis, that were increased only at CSCs after compound **1** treatment, includes GalNAc addition. GalNAc residue must be activated by binding to UDP-GalNAc. That is achieved mostly by conversion of UDP-GlcNAc to UDP-GalNAc (Fig. 9B). Fructose-6-P is common metabolite of glycolysis and UDP-GalNAc synthesis. Neu5Ac, needed for acidic GSL synthesis, and UDP-GalNAc share UDP-GlcNAc as common precursor (Fig. 9B)^30^. In non-treated MDA-MB-231 cells, we found a 23-fold higher GalNAc-GM1b expression in non-CSCs compared to CSCs, together with increased Gb_4_Cer and IV^6^Neu5Ac-nLc_4_Cer, all containing GalNAc as last sugar residue (Fig. 9A, C). In addition, gangliosides GM2 and GM3, were increased in non-treated non-CSCs, but not so dramatically as GalNAc-GM1b. These results indicate that glycolysis could be slower in non-treated non-CSCs in comparison to CSC, giving more precursors for UDP-GalNAc and Neu5Ac synthesis. Therefore, the findings at CSCs after compound **1** treatment of MDA-MB-231 cells, increased GM3, IV^6^Neu5Ac-nLc_4_Cer, GalNAc-GM1b, Gb_4_Cer, and GM2, could indicate CSC glycolysis slowdown. Cancer stem cells of glioma are more glycolytic than non-CSCs due to a mitochondrial voltage-dependent anion channel that controls the phenotype transition between glioma stem cells and non-stem cells^31^. The channel is highly expressed in non-CSC relative to CSC and coupled to a glycolytic rate-limiting enzyme platelet-type of phosphofructokinase on mitochondrion to inhibit kinase-mediated glycolysis required for CSC maintenance.

During tumorigensis, distinct GalNAc transferases (GALNTs) can be differently expressed. Glycosylation of E-cadherin with GalNAc starts in the Golgi apparatus by glycosyltransferases called GALNTs^32^. E-cadherin combines mechanotransduction and EGFR signaling to regulate junctional tissue polarization and tight junction positioning^33^, GALNT3 preserves the epithelial state in trophoblast stem cells. The loss of GALNT3 expression diminishes O-GalNAc glycosylation and causes epithelial–mesenchymal transition^32^. Due to replacement of E-cadherin by N-cadherin in the mammary gland, fibrocystic changes and tumor formation occur^34^. N-cadherin causes FGFR upmodulation which results in epithelial-to-mesenchymal transition (EMT) and stem/progenitor like properties^35^. We can speculate that GALNT responsible for GalNAc-GM1b synthesis is sensitive to similar effectors as GALNT3. There is no data in literature concerning GalNAc-GM1b expression in cancer stem cells. We found lower GalNAc-GM1b in CSCs compared to non-CSC MDA-MB231. This finding is in accordance with the results of Guan et al. During induced epithelial–mesenchymal transition of breast cells, there is significantly reduced Gg4 and its synthase B3GALT4^19^. Neutral glycosphingolipid Gg4 is a direct precursor of GM1b. GM1b is further a direct precursor of GalNAc-GM1b, as it is presented in Fig. 9A. On the other hand, GALNT14 shows the opposite effects. It catalyzes O-glycosylation of EGF-containing fibulin-like extracellular matrix protein 2. This significantly increases the invasion ability of breast cancer cell lines (MCF-7 and MBA-MD-231)^36,37^. GALNT14 is related to the chemosensitivity of breast cancer. Osterix, a zinc finger-containing transcription factor, decreases chemosensitivity and enhances anti-apoptosis by upregulating GALNT14^38^. Osterix has also important roles in facilitating breast cancer invasion^39^.

In our study, treated CSCs acquired a phenotype closer to non-treated non-CSCs. Malignancy is not only defined by tumour-specific molecules, or their genes, but it can be caused by disorganization of cell membrane components^40^. Glycosphingolipids are important cell membrane components being able to influence final cell behaviour. Gb3Cer plays an essential role in the maintenance of epithelial cancer cell properties. Depletion of Gb3Cer by deletion of the key enzyme lactosylceramide 4-alpha-galactosyltransferase (A4GALT) induces epithelial-to-mesenchymal transition, enhances chemoresistance, and increases CD44^+^/CD24^−^ cells^41^. The cholera toxin-induced mesenchymal-to-epithelial transition occurred only in cells with functional A4GALT. Cholera toxin is able to induce transition after binding to its receptor, Gb3Cer^42^. Liang et al*.* described greatly reduced levels of Gb3Cer in breast CSCs in comparison to cancer non-stem cells (non-CSCs)^15^. Whilst we did not determine Gb3Cer, the enzyme A4GALT was obviously active in our study because Gb3Cer is direct precursor of Gb_4_Cer and Gb_4_Cer was found elevated in CSCs after treatment with compound **1**^42^. We have not found elevated GM2 and GD3 in CSCs as was earlier reported^15^. Our study used MDA-MB-231 and MDA-MB-453 breast CSCs and non-CSCs while Liang et al*.* used a model of epithelial–mesenchymal transition induction of immortalized human mammary epithelial cell–Twist-estrogen receptor (HMLE-Twist-ER)^15^. They proved increased GM2 and GD3 in organic solvent extract of GSLs using methods of Orbitrap-Fourier transform (FT) mass spectrometry (MS) and high-performance liquid thin layer chromatography (HPTLC)-immunostaining. That means, GSLs from the plasma membrane and from Golgi are included in final findings. Their flow-cytometry results have not proved elevated GM2 and CSCs. The percentage of GD3 was reported as elevated, but due to their gating strategy, they had excluded the most GD3 positive cells from analyses of their CSC markers (CD44^+^/CD24^−^)^15^. Therefore, our final results are not comparable.

Sialylation is involved in cell fate decision during development, reprogramming and cancer progression^43^. Sialylated GM3 and IV^6^Neu5Ac-nLc_4_Cer were increased in CSCs after compound **1** treatment of both MDA-MB-231 and MDA-MB-453 cell lines. Ganglioside GM3 is typically located in specialized membrane microdomains called lipid-rafts^44^. Enhanced GM3 lipid raft content disturbs insulin receptor function causing insulin resistance and finally diabetes type 2^45^. In a similar manner, change of the GM3 content in cell membranes could influence co-localized fibroblast growth factor (FGF) receptor action, that is involved in cancer pathogenesis^46^: low level of GM3 activates and high level inhibits FGF signal transduction^47^. Therefore, we could assume that increased GM3 in CSCs, after treatment with compound **1**, contributes to inhibition of FGF signaling and thereby reduces cancer progression. Sialylation of the nLc_4_Cer to form IV^6^Neu5Ac-nLc_4_Cer is catalysed by enzyme sialyl transferase to nLc_4_Cer (ST6Gal). An increase in infiltrating lymphocytes is influenced by high expression of ST6Gal-II in triple negative breast cancers that correspond to our breast cancer model^48^. Triple negative breast cancer lymphocyte infiltration correlates with better overall survival and better chemotherapeutic responses^49^.

Concerning findings in MDA-MB-453 cells, only increased percent of both GalNAc-GM1b^+^CD44^+^/CD24^−^ (data not shown) and GalNAc-GM1b^+^CD133^+^ cells after compound **1** treatment (Fig. 7A) corresponded to MDA-MB-231 GSL findings. Yang et al., detected 31 patients containing CSCs among 88 primary TNBCs, using CD44^+^/CD24^−^, aldehyde dehydrogenase family 1 member A1 (ALDH1A1) and CD133 markers. Eight cases were positive for both CD44^+^/CD24^−^ and ALDH1A1, 10 cases were positive for both CD44^+^/CD24^−^ and CD133, 9 cases were positive for both ALDH1A1 and CD133, while only 4 cases showed positivity of all the three CSC markers^50^. Correlation between CSC markers was weak, implying that most breast cancer cells do not express these markers concurrently. Knowing that CD133 phenotype is not significantly associated with worse progression-free survival, we can assume that CD133 is less reliable CSC marker compared to CD44^+^/CD24^−^^50^. Expression of MDA-MB-231 glycosphingolipids found in this study could have higher impact for clinical implications. Dramatically reduction of GalNAc glycosylation observed in MDA-MB-231 CSCs after compound **1** treatment, characterized earlier to be enrolled in either epithelial–mesenchymal transition or in its reversal, could indicate possible biochemical pathway of CSC reduction by compound **1**.

## Conclusions

The novel thieno[2,3-*b*]pyridine anticancer compound **1** was cytotoxic for the breast cancer cells, cell death being mediated by apoptosis. The percent of cancer stem cells was significantly lower. Glycosphingolipids IV^6^Neu5Ac-nLc_4_Cer and GalNAc-GM1b, not reported previously, were identified in both breast cancer stem cells and cancer non-stem cells. IV^6^Neu5Ac-nLc_4_Cer had increased expression in both cancer stem cells and cancer non-stem cells of both MDA-MB-231 and MDA-MB-453 cell lines after treatment with compound **1**, while GM3 had increased expression only on both cell subpopulations in MDA-MB-231 cell line. GalNAc-GM1b, Gb_4_Cer and GM2 were increased only in cancer stem cells of both cell lines while GD3 was decreased in cancer non-stem cells of MDA-MB-231 cell line, 48 h after treatment with **1**.

Due to the demonstrated effect in reducing the percentage of cancer stem cells and number of mammospheres and the shift of CSC to non-CSC glycophenotype, the novel thieno[2,3-*b*]pyridine anticancer compound **1** deserves attention as a potential new drug for triple-negative breast cancer therapy.

## Data Availability

The datasets used and/or analyzed during the current study are available from the corresponding author on reasonable request.
