# Author Correction: Glycosphingolipid expression at breast cancer stem cells after novel thieno[2,3-*b*]pyridine anticancer compound treatment

**DOI:** 10.1038/s41598-021-87310-y

**Published:** 2021-04-09

**Authors:** Sandra Marijan, Anita Markotić, Angela Mastelić, Nikolina Režić-Mužinić, Lisa Ivy Pilkington, Johannes Reynisson, Vedrana Čikeš Čulić

**Affiliations:** 1 Department of Medical Chemistry and Biochemistry, University of Split School of Medicine, 21000 Split, Croatia; 2 School of Chemical Sciences, The University of Auckland, Auckland, New Zealand; 3 School of Pharmacy and Bioengineering, Keele University, Staffordshire, UK

Correction to: *Scientific Reports* https://doi.org/10.1038/s41598-020-68516-y, published online 17 July 2020

The Author Contributions section in this Article was incomplete.

“S.M. performed tissue culture and flow cytometric studies, participated in design of experiments and interpreted the data; A.M. conceptualization and design of experiments, data analysis and interpretation, major part in manuscript writing; A.M. performed tissue culture and flow cytometric studies, performed data analysis; N.R.M. performed tissue culture and flow cytometric studies; L.I.P. performed design and synthesis of chemical compounds, writing and critical reading of manuscript; J.R. performed design and synthesis of chemical compounds, conceptualization of experiments, writing and critical reading of manuscript and data interpretation; V.Č.Č. conceptualization and design of experiments, data analysis and interpretation and writing of manuscript. All authors read and approved the final manuscript.”

now reads:

“S.M. performed tissue culture and flow cytometric studies, participated in design of experiments, interpretation of the data, writing and critical reading of the manuscript; A.M. conceptualization and design of experiments, data analysis and interpretation, major part in manuscript writing; A.M. performed tissue culture and flow cytometric studies, performed data analysis; N.R.M. performed tissue culture and flow cytometric studies; L.I.P. performed design and synthesis of chemical compounds, writing and critical reading of manuscript; J.R. performed design and synthesis of chemical compounds, conceptualization of experiments, writing and critical reading of manuscript and data interpretation; V.Č.Č. conceptualization and design of experiments, data analysis and interpretation and writing of manuscript. All authors read and approved the final manuscript.”

The original Article has been corrected.

